# 3-Hydroxy­meth­yl-6,8-dimeth­oxy-2*H*-chromen-2-one

**DOI:** 10.1107/S1600536808015833

**Published:** 2008-06-07

**Authors:** Mei-yan Wei, Zhen Liu, Chang-lun Shao, Zhen-bin Jia, Chang-yun Wang

**Affiliations:** a School of Pharmacy, Guangdong Medical College, Dongguan, Guangdong 523808, People’s Republic of China; b College of Chemistry and Chemical Engineering, Luoyang Normal University, Luoyang, Henan 471022, People’s Republic of China; c School of Medicine and Pharmacy, Ocean University of China, Qingdao, Shandong 266003, People’s Republic of China

## Abstract

The asymmetric unit of the title compound, C_12_H_12_O_5_, contains four independent mol­ecules. In the crystal structure, inter­molecular O—H⋯O hydrogen bonds link the mol­ecules into one-dimensional infinite chains. They are arranged in a nearly parallel fashion along the *b* axis and stabilized by π–π inter­actions [3.443 (2) Å].

## Related literature

For related literature, see: Ayer *et al.* (1990 ▶).
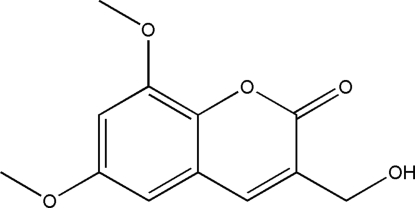


## Experimental

### 

#### Crystal data

C_12_H_12_O_5_
*M* *_r_* = 236.22
Monoclinic, 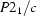

*a* = 14.6979 (16) Å
*b* = 12.2246 (14) Å
*c* = 23.896 (3) Å
β = 95.035 (2)°
*V* = 4277.0 (9) Å^3^
*Z* = 16
Mo *K*α radiation
μ = 0.12 mm^−1^
*T* = 173 (2) K
0.49 × 0.42 × 0.25 mm

#### Data collection

Bruker SMART CCD area-detector diffractometer
Absorption correction: multi-scan (*SADABS*; Bruker, 2001 ▶) *T*_min_ = 0.946, *T*_max_ = 0.972
19027 measured reflections
7927 independent reflections
4460 reflections with *I* > 2σ(*I*)
*R*_int_ = 0.041

#### Refinement

*R*[*F* ^2^ > 2σ(*F* ^2^)] = 0.054
*wR*(*F* ^2^) = 0.170
*S* = 1.00
7927 reflections
625 parameters
H-atom parameters constrained
Δρ_max_ = 0.55 e Å^−3^
Δρ_min_ = −0.34 e Å^−3^

### 

Data collection: *SMART* (Bruker, 1997 ▶); cell refinement: *SAINT* (Bruker, 2001 ▶); data reduction: *SAINT*; program(s) used to solve structure: *SHELXS97* (Sheldrick, 2008 ▶); program(s) used to refine structure: *SHELXL97* (Sheldrick, 2008 ▶); molecular graphics: *SHELXTL* (Sheldrick, 2008 ▶); software used to prepare material for publication: *SHELXTL*.

## Supplementary Material

Crystal structure: contains datablocks global, I. DOI: 10.1107/S1600536808015833/hk2466sup1.cif

Structure factors: contains datablocks I. DOI: 10.1107/S1600536808015833/hk2466Isup2.hkl

Additional supplementary materials:  crystallographic information; 3D view; checkCIF report

## Figures and Tables

**Table 1:** Hydrogen-bond geometry (Å, °)

*D*—H⋯*A*	*D*—H	H⋯*A*	*D*⋯*A*	*D*—H⋯*A*
O20—H20⋯O2^i^	0.84	2.00	2.829 (2)	171
O15—H15⋯O12^ii^	0.84	1.96	2.800 (2)	175
O10—H10⋯O7^iii^	0.84	2.00	2.838 (2)	174
O5—H5⋯O17^iv^	0.84	1.96	2.790 (2)	173

Symmetry codes: (i) 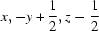; (ii) 
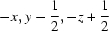
; (iii) 
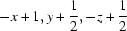
; (iv) 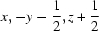.

**Fig. 1.**
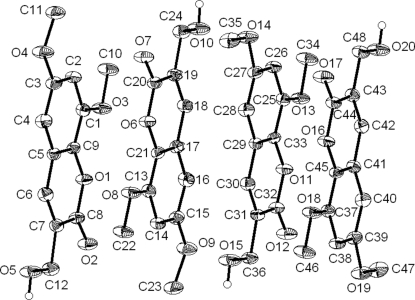
The molecular structure of the title molecule, with the atom-numbering scheme. Displacement ellipsoids are drawn at the 30% probability level. H atoms are omitted for clarity.

**Fig. 2.**
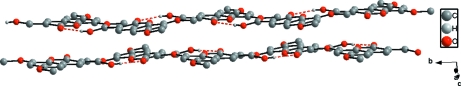
A partial packing diagram of (I). Hydrogen bonds are shown as dashed lines.

## supplementary crystallographic information

### Comment

The title compound was isolated from the endophytic fungus No. 2090 of the mangrove tree from the South China Sea coast. This substance was previously isolated from the organic extracts of the fungus Verticilliumdahliae Kleb (Ayer et al., 1978). However, the structure of the title compound, (I), was previously elucidated on the basic of spectroscopic analysis. For further confirming the analytical results, we report herein its crystal structure.

The asymmetric unit of (I) (Fig. 1) contains four independent molecules.

In the crystal structure, intermolecular O—H···O hydrogen bonds (Table 1) link the molecules into one-dimensional infinite chains (Fig. 2). Morover, they are arranged in nearly parallel fashion along the b axis and stabilized by the π–π interactions.

### Experimental

A strain of fungus (No. 2090) was deposited in the Department of Applied Chemistry, Zhongshan University, Guangzhou, People's Republic of China. Culture conditions: GYT medium (glucose 10 g /*L*, peptone 2 g /*L*, yeast extract 1 g /*L*, NaCl 2.5 g /*L*) and incubation at 298 K for 24 d. For the extraction and separation of the metabolite, the cultures (70 *L*) of the title compound were filtered through cheesecloth. The filtrate was concentrated to 3 *L* below 323 K, then extracted five times by shaking with an equal volume of ethyl acetate. The extract was evaporated under reduced pressure below 323 K. The combined organic extracts were subjected to silica-gel column chromatography, eluting with petroleum ether/ethyl acetate, to yield the title compound. Crystals were obtained through evaporation of a methanol solution.

### Refinement

H atoms were positioned geometrically, with O—H = 0.84 Å (for OH) and C—H = 0.95, 0.99 and 0.98 Å for aromatic methylene and methyl H, respectively, and constrained to ride on their parent atoms with U_iso_(H) = xU_eq_(C,O), where x = 1.5 for OH H, and x = 1.2 for all other H atoms.

### Crystal data

**Table:**

C_12_H_12_O_5_	*F*_000_ = 1984
*M**_r_* = 236.22	*D*_x_ = 1.467 Mg m^−^^3^
Monoclinic, *P*2_1_/*c*	Mo *K*α radiation λ = 0.71073 Å
Hall symbol: -P 2ybc	Cell parameters from 5413 reflections
*a* = 14.6979 (16) Å	θ = 2.2–27.0º
*b* = 12.2246 (14) Å	µ = 0.12 mm^−^^1^
*c* = 23.896 (3) Å	*T* = 173 (2) K
β = 95.035 (2)º	Block, yellow
*V* = 4277.0 (9) Å^3^	0.49 × 0.42 × 0.25 mm
*Z* = 16	

### Data collection

**Table:**

Bruker SMART CCD area-detector diffractometer	7927 independent reflections
Radiation source: fine-focus sealed tube	4460 reflections with *I* > 2σ(*I*)
Monochromator: graphite	*R*_int_ = 0.041
*T* = 173(2) K	θ_max_ = 25.5º
φ and ω scans	θ_min_ = 1.7º
Absorption correction: multi-scan(SADABS; Bruker, 2001)	*h* = −17→16
*T*_min_ = 0.946, *T*_max_ = 0.972	*k* = −6→14
19027 measured reflections	*l* = −28→28

### Refinement

**Table:**

Refinement on *F*^2^	Secondary atom site location: difference Fourier map
Least-squares matrix: full	Hydrogen site location: inferred from neighbouring sites
*R*[*F*^2^ > 2σ(*F*^2^)] = 0.054	H-atom parameters constrained
*wR*(*F*^2^) = 0.170	*w* = 1/[σ^2^(*F*_o_^2^) + (0.0977*P*)^2^] where *P* = (*F*_o_^2^ + 2*F*_c_^2^)/3
*S* = 1.00	(Δ/σ)_max_ < 0.001
7927 reflections	Δρ_max_ = 0.55 e Å^−^^3^
625 parameters	Δρ_min_ = −0.34 e Å^−^^3^
Primary atom site location: structure-invariant direct methods	Extinction correction: none

### Special details

**Table:**

Geometry. All e.s.d.'s (except the e.s.d. in the dihedral angle between two l.s. planes) are estimated using the full covariance matrix. The cell e.s.d.'s are taken into account individually in the estimation of e.s.d.'s in distances, angles and torsion angles; correlations between e.s.d.'s in cell parameters are only used when they are defined by crystal symmetry. An approximate (isotropic) treatment of cell e.s.d.'s is used for estimating e.s.d.'s involving l.s. planes.
Refinement. Refinement of F^2^ against ALL reflections. The weighted R-factor wR and goodness of fit S are based on F^2^, conventional R-factors R are based on F, with F set to zero for negative F^2^. The threshold expression of F^2^ > 2sigma(F^2^) is used only for calculating R-factors(gt) etc. and is not relevant to the choice of reflections for refinement. R-factors based on F^2^ are statistically about twice as large as those based on F, and R- factors based on ALL data will be even larger.

### Fractional atomic coordinates and isotropic or equivalent isotropic displacement parameters (Å^2^)

**Table:**

	*x*	*y*	*z*	*U*_iso_*/*U*_eq_	
O1	0.34046 (10)	0.06130 (12)	0.46126 (6)	0.0222 (4)	
O2	0.23122 (10)	−0.02976 (13)	0.49764 (7)	0.0292 (4)	
O3	0.39758 (10)	0.25128 (12)	0.43110 (7)	0.0285 (4)	
O4	0.66666 (11)	0.07960 (14)	0.36495 (7)	0.0357 (5)	
O5	0.36461 (10)	−0.32898 (13)	0.47854 (8)	0.0363 (5)	
H5	0.3374	−0.3868	0.4863	0.054*	
O6	0.40867 (10)	0.01426 (12)	0.28271 (6)	0.0232 (4)	
O7	0.51833 (10)	0.10466 (13)	0.24619 (7)	0.0303 (4)	
O8	0.34984 (10)	−0.17613 (12)	0.31126 (7)	0.0289 (4)	
O9	0.08454 (10)	−0.00244 (14)	0.38154 (7)	0.0345 (4)	
O10	0.38760 (11)	0.40517 (13)	0.26800 (8)	0.0368 (5)	
H10	0.4159	0.4627	0.2612	0.055*	
O11	0.07893 (9)	0.13556 (12)	0.21061 (6)	0.0223 (4)	
O12	−0.02891 (10)	0.04463 (13)	0.24848 (7)	0.0302 (4)	
O13	0.13692 (10)	0.32770 (12)	0.18371 (7)	0.0263 (4)	
O14	0.40080 (10)	0.17646 (13)	0.11225 (7)	0.0318 (4)	
O15	0.10756 (10)	−0.25362 (13)	0.22829 (8)	0.0368 (5)	
H15	0.0815	−0.3121	0.2362	0.055*	
O16	0.16996 (10)	−0.06143 (12)	0.03306 (6)	0.0237 (4)	
O17	0.27877 (10)	0.02844 (13)	−0.00470 (7)	0.0311 (4)	
O18	0.10944 (10)	−0.25392 (12)	0.05812 (7)	0.0276 (4)	
O19	−0.15022 (10)	−0.09995 (13)	0.13372 (7)	0.0312 (4)	
O20	0.14506 (11)	0.32841 (13)	0.01782 (8)	0.0356 (5)	
H20	0.1698	0.3864	0.0079	0.053*	
C1	0.45277 (14)	0.16623 (18)	0.42108 (9)	0.0207 (5)	
C2	0.53456 (15)	0.17311 (19)	0.39616 (9)	0.0234 (5)	
H2	0.5560	0.2420	0.3844	0.028*	
C3	0.58498 (14)	0.07853 (19)	0.38857 (10)	0.0244 (6)	
C4	0.55564 (15)	−0.0224 (2)	0.40567 (10)	0.0266 (6)	
H4	0.5914	−0.0859	0.4009	0.032*	
C5	0.47303 (14)	−0.03036 (19)	0.43007 (9)	0.0211 (5)	
C6	0.43561 (15)	−0.13227 (19)	0.44780 (9)	0.0237 (5)	
H6	0.4687	−0.1981	0.4437	0.028*	
C7	0.35488 (15)	−0.13599 (18)	0.47000 (9)	0.0222 (5)	
C8	0.30436 (15)	−0.03491 (18)	0.47759 (9)	0.0221 (5)	
C9	0.42242 (14)	0.06384 (18)	0.43726 (9)	0.0196 (5)	
C10	0.43098 (16)	0.35850 (18)	0.42034 (11)	0.0313 (6)	
H10A	0.4897	0.3699	0.4423	0.047*	
H10B	0.3870	0.4132	0.4311	0.047*	
H10C	0.4392	0.3659	0.3803	0.047*	
C11	0.69700 (16)	0.1788 (2)	0.34201 (11)	0.0332 (6)	
H11A	0.6495	0.2072	0.3145	0.050*	
H11B	0.7529	0.1652	0.3236	0.050*	
H11C	0.7095	0.2324	0.3722	0.050*	
C12	0.30893 (15)	−0.23834 (18)	0.48782 (10)	0.0265 (6)	
H12A	0.2987	−0.2337	0.5281	0.032*	
H12B	0.2489	−0.2467	0.4660	0.032*	
C13	0.29574 (15)	−0.09025 (19)	0.32277 (9)	0.0215 (5)	
C14	0.21541 (14)	−0.09679 (19)	0.34822 (9)	0.0229 (5)	
H14	0.1940	−0.1658	0.3598	0.027*	
C15	0.16535 (15)	−0.00206 (19)	0.35712 (9)	0.0242 (5)	
C16	0.19569 (15)	0.09855 (19)	0.34029 (9)	0.0251 (5)	
H16	0.1607	0.1624	0.3457	0.030*	
C17	0.27796 (14)	0.10636 (18)	0.31530 (9)	0.0207 (5)	
C18	0.31568 (15)	0.20840 (18)	0.29729 (9)	0.0229 (5)	
H18	0.2832	0.2745	0.3019	0.027*	
C19	0.39549 (14)	0.21186 (18)	0.27420 (9)	0.0217 (5)	
C20	0.44543 (15)	0.11029 (18)	0.26644 (9)	0.0221 (5)	
C21	0.32695 (14)	0.01213 (18)	0.30696 (9)	0.0199 (5)	
C22	0.31769 (17)	−0.28333 (18)	0.32396 (11)	0.0304 (6)	
H22A	0.3139	−0.2900	0.3646	0.046*	
H22B	0.3601	−0.3384	0.3117	0.046*	
H22C	0.2571	−0.2949	0.3043	0.046*	
C23	0.05554 (16)	−0.1015 (2)	0.40576 (10)	0.0319 (6)	
H23A	0.0453	−0.1572	0.3764	0.048*	
H23B	−0.0014	−0.0886	0.4232	0.048*	
H23C	0.1027	−0.1268	0.4343	0.048*	
C24	0.44016 (15)	0.31340 (18)	0.25490 (10)	0.0268 (6)	
H24A	0.5026	0.3198	0.2738	0.032*	
H24B	0.4447	0.3099	0.2138	0.032*	
C25	0.19106 (14)	0.24339 (18)	0.17149 (9)	0.0203 (5)	
C26	0.27178 (14)	0.25041 (18)	0.14697 (9)	0.0222 (5)	
H26	0.2938	0.3199	0.1366	0.027*	
C27	0.32196 (14)	0.15630 (19)	0.13717 (9)	0.0224 (5)	
C28	0.29236 (15)	0.05458 (19)	0.15189 (9)	0.0241 (5)	
H28	0.3272	−0.0088	0.1451	0.029*	
C29	0.20983 (14)	0.04547 (18)	0.17709 (9)	0.0192 (5)	
C30	0.17217 (15)	−0.05651 (19)	0.19477 (9)	0.0220 (5)	
H30	0.2044	−0.1225	0.1894	0.026*	
C31	0.09317 (15)	−0.06079 (18)	0.21847 (9)	0.0205 (5)	
C32	0.04357 (15)	0.03915 (18)	0.22739 (9)	0.0216 (5)	
C33	0.16038 (14)	0.13937 (18)	0.18639 (9)	0.0185 (5)	
C34	0.17076 (17)	0.43486 (18)	0.17191 (11)	0.0335 (6)	
H34A	0.2298	0.4464	0.1935	0.050*	
H34B	0.1272	0.4902	0.1825	0.050*	
H34C	0.1784	0.4410	0.1317	0.050*	
C35	0.45447 (15)	0.0838 (2)	0.09976 (11)	0.0338 (6)	
H35A	0.4754	0.0460	0.1347	0.051*	
H35B	0.5075	0.1080	0.0808	0.051*	
H35C	0.4174	0.0337	0.0752	0.051*	
C36	0.05020 (15)	−0.16391 (18)	0.23784 (10)	0.0238 (5)	
H36A	0.0415	−0.1585	0.2784	0.029*	
H36B	−0.0104	−0.1746	0.2170	0.029*	
C37	0.05696 (15)	−0.16841 (19)	0.07157 (9)	0.0212 (5)	
C38	−0.02323 (14)	−0.17498 (18)	0.09693 (9)	0.0228 (5)	
H38	−0.0458	−0.2445	0.1070	0.027*	
C39	−0.07202 (15)	−0.08039 (19)	0.10811 (9)	0.0234 (5)	
C40	−0.04122 (14)	0.02177 (19)	0.09371 (9)	0.0230 (5)	
H40	−0.0749	0.0857	0.1011	0.028*	
C41	0.04095 (15)	0.02945 (18)	0.06786 (9)	0.0209 (5)	
C42	0.07907 (15)	0.13177 (18)	0.05071 (9)	0.0217 (5)	
H42	0.0479	0.1981	0.0571	0.026*	
C43	0.15768 (15)	0.13529 (18)	0.02582 (9)	0.0220 (5)	
C44	0.20614 (15)	0.03467 (18)	0.01684 (9)	0.0226 (5)	
C45	0.08888 (14)	−0.06456 (18)	0.05764 (9)	0.0189 (5)	
C46	0.07568 (17)	−0.36016 (19)	0.07114 (11)	0.0337 (6)	
H46A	0.0718	−0.3661	0.1118	0.051*	
H46B	0.1173	−0.4163	0.0590	0.051*	
H46C	0.0149	−0.3707	0.0516	0.051*	
C47	−0.20234 (15)	−0.0070 (2)	0.14792 (10)	0.0321 (6)	
H47A	−0.1643	0.0410	0.1732	0.048*	
H47B	−0.2555	−0.0312	0.1667	0.048*	
H47C	−0.2230	0.0332	0.1136	0.048*	
C48	0.19980 (15)	0.23813 (18)	0.00552 (10)	0.0250 (5)	
H48A	0.2621	0.2472	0.0243	0.030*	
H48B	0.2044	0.2336	−0.0355	0.030*	

### Atomic displacement parameters (Å^2^)

**Table:**

	*U*^11^	*U*^22^	*U*^33^	*U*^12^	*U*^13^	*U*^23^
O1	0.0190 (8)	0.0156 (8)	0.0336 (9)	−0.0001 (7)	0.0122 (7)	0.0015 (7)
O2	0.0240 (9)	0.0185 (9)	0.0475 (11)	0.0006 (7)	0.0170 (8)	0.0021 (8)
O3	0.0263 (9)	0.0137 (9)	0.0479 (11)	0.0007 (7)	0.0169 (8)	0.0038 (8)
O4	0.0277 (9)	0.0263 (10)	0.0566 (12)	0.0021 (8)	0.0237 (9)	0.0079 (9)
O5	0.0302 (10)	0.0146 (9)	0.0670 (13)	0.0002 (7)	0.0210 (9)	0.0051 (9)
O6	0.0210 (8)	0.0162 (9)	0.0339 (9)	−0.0007 (7)	0.0106 (7)	0.0017 (7)
O7	0.0260 (9)	0.0186 (9)	0.0488 (11)	−0.0012 (7)	0.0178 (8)	0.0042 (8)
O8	0.0275 (9)	0.0134 (9)	0.0483 (11)	−0.0004 (7)	0.0169 (8)	0.0031 (8)
O9	0.0260 (9)	0.0300 (10)	0.0502 (11)	0.0032 (8)	0.0202 (8)	0.0090 (8)
O10	0.0354 (10)	0.0147 (9)	0.0635 (12)	0.0001 (8)	0.0221 (9)	0.0041 (9)
O11	0.0204 (8)	0.0139 (8)	0.0344 (9)	0.0001 (7)	0.0122 (7)	0.0020 (7)
O12	0.0277 (9)	0.0187 (9)	0.0472 (11)	−0.0012 (7)	0.0203 (8)	0.0012 (8)
O13	0.0247 (8)	0.0108 (8)	0.0455 (10)	0.0016 (7)	0.0145 (8)	0.0046 (7)
O14	0.0248 (9)	0.0214 (9)	0.0524 (11)	0.0009 (7)	0.0219 (8)	0.0009 (8)
O15	0.0314 (9)	0.0149 (9)	0.0673 (12)	−0.0003 (8)	0.0214 (9)	0.0065 (9)
O16	0.0238 (8)	0.0149 (8)	0.0340 (9)	0.0000 (7)	0.0124 (7)	0.0035 (7)
O17	0.0298 (9)	0.0193 (9)	0.0473 (11)	0.0009 (8)	0.0207 (8)	0.0030 (8)
O18	0.0274 (9)	0.0120 (8)	0.0460 (11)	0.0019 (7)	0.0172 (8)	0.0044 (7)
O19	0.0244 (9)	0.0218 (9)	0.0505 (11)	0.0009 (7)	0.0203 (8)	−0.0017 (8)
O20	0.0344 (10)	0.0144 (9)	0.0614 (12)	0.0007 (8)	0.0230 (9)	0.0052 (9)
C1	0.0206 (12)	0.0180 (12)	0.0241 (13)	0.0029 (10)	0.0052 (10)	0.0010 (10)
C2	0.0237 (12)	0.0186 (13)	0.0290 (13)	−0.0028 (10)	0.0079 (10)	0.0026 (10)
C3	0.0183 (12)	0.0239 (13)	0.0325 (14)	0.0020 (10)	0.0107 (10)	0.0012 (11)
C4	0.0231 (12)	0.0195 (13)	0.0380 (15)	0.0029 (10)	0.0084 (11)	−0.0001 (11)
C5	0.0199 (12)	0.0189 (12)	0.0245 (13)	−0.0007 (10)	0.0028 (10)	0.0010 (10)
C6	0.0250 (13)	0.0148 (12)	0.0316 (14)	0.0027 (10)	0.0037 (11)	−0.0012 (10)
C7	0.0223 (12)	0.0178 (12)	0.0265 (13)	−0.0002 (10)	0.0025 (10)	0.0013 (10)
C8	0.0231 (12)	0.0158 (12)	0.0279 (13)	−0.0038 (10)	0.0055 (10)	−0.0009 (10)
C9	0.0160 (11)	0.0193 (13)	0.0239 (13)	0.0009 (10)	0.0037 (10)	0.0002 (10)
C10	0.0361 (14)	0.0123 (12)	0.0479 (16)	−0.0015 (11)	0.0165 (12)	0.0011 (11)
C11	0.0271 (13)	0.0309 (15)	0.0439 (16)	−0.0025 (11)	0.0160 (12)	0.0065 (12)
C12	0.0248 (12)	0.0164 (12)	0.0391 (15)	0.0008 (10)	0.0074 (11)	0.0007 (11)
C13	0.0225 (12)	0.0175 (12)	0.0249 (13)	0.0026 (10)	0.0043 (10)	0.0014 (10)
C14	0.0239 (12)	0.0183 (12)	0.0272 (13)	−0.0035 (10)	0.0058 (10)	0.0023 (10)
C15	0.0185 (12)	0.0279 (14)	0.0271 (13)	−0.0002 (10)	0.0075 (10)	0.0003 (10)
C16	0.0251 (12)	0.0186 (13)	0.0325 (14)	0.0063 (10)	0.0083 (11)	0.0012 (10)
C17	0.0198 (11)	0.0173 (12)	0.0250 (13)	0.0016 (10)	0.0025 (10)	0.0001 (10)
C18	0.0272 (12)	0.0136 (12)	0.0280 (13)	0.0008 (10)	0.0033 (10)	0.0006 (10)
C19	0.0231 (12)	0.0163 (12)	0.0260 (13)	−0.0014 (10)	0.0033 (10)	0.0017 (10)
C20	0.0210 (12)	0.0178 (12)	0.0279 (13)	−0.0031 (10)	0.0040 (10)	0.0015 (10)
C21	0.0176 (11)	0.0194 (13)	0.0231 (13)	−0.0002 (10)	0.0034 (10)	−0.0001 (10)
C22	0.0359 (14)	0.0125 (12)	0.0450 (16)	−0.0024 (11)	0.0164 (12)	0.0026 (11)
C23	0.0259 (13)	0.0317 (15)	0.0402 (16)	−0.0037 (11)	0.0154 (12)	0.0022 (12)
C24	0.0260 (13)	0.0168 (12)	0.0387 (15)	0.0015 (10)	0.0093 (11)	0.0013 (11)
C25	0.0221 (11)	0.0157 (12)	0.0234 (12)	0.0021 (10)	0.0039 (10)	0.0007 (10)
C26	0.0212 (11)	0.0157 (12)	0.0305 (13)	−0.0013 (10)	0.0071 (10)	0.0013 (10)
C27	0.0179 (11)	0.0214 (12)	0.0287 (13)	−0.0003 (10)	0.0072 (10)	0.0006 (10)
C28	0.0225 (12)	0.0181 (13)	0.0326 (14)	0.0030 (10)	0.0075 (11)	−0.0003 (10)
C29	0.0191 (12)	0.0166 (12)	0.0222 (12)	−0.0006 (10)	0.0045 (10)	0.0018 (10)
C30	0.0241 (12)	0.0146 (12)	0.0275 (13)	0.0021 (10)	0.0045 (10)	−0.0002 (10)
C31	0.0226 (12)	0.0153 (12)	0.0238 (12)	−0.0040 (10)	0.0038 (10)	0.0010 (10)
C32	0.0220 (12)	0.0179 (12)	0.0257 (13)	−0.0036 (10)	0.0071 (10)	0.0011 (10)
C33	0.0150 (11)	0.0178 (12)	0.0229 (13)	−0.0017 (9)	0.0032 (9)	0.0013 (10)
C34	0.0382 (15)	0.0110 (12)	0.0544 (17)	0.0029 (11)	0.0217 (13)	0.0051 (11)
C35	0.0239 (13)	0.0257 (14)	0.0547 (18)	0.0057 (11)	0.0205 (12)	−0.0023 (12)
C36	0.0224 (12)	0.0152 (12)	0.0349 (14)	−0.0005 (10)	0.0079 (11)	0.0027 (10)
C37	0.0209 (12)	0.0169 (12)	0.0261 (13)	0.0018 (10)	0.0042 (10)	0.0022 (10)
C38	0.0229 (12)	0.0152 (12)	0.0313 (14)	−0.0036 (10)	0.0080 (10)	−0.0003 (10)
C39	0.0195 (12)	0.0211 (13)	0.0303 (14)	0.0002 (10)	0.0063 (10)	−0.0015 (10)
C40	0.0226 (12)	0.0167 (12)	0.0304 (14)	0.0040 (10)	0.0070 (10)	−0.0028 (10)
C41	0.0234 (12)	0.0172 (12)	0.0222 (12)	0.0001 (10)	0.0036 (10)	0.0012 (10)
C42	0.0233 (12)	0.0154 (12)	0.0268 (13)	0.0039 (10)	0.0033 (10)	−0.0016 (10)
C43	0.0234 (12)	0.0183 (13)	0.0247 (13)	0.0015 (10)	0.0041 (10)	0.0013 (10)
C44	0.0239 (12)	0.0167 (12)	0.0280 (13)	−0.0018 (10)	0.0063 (10)	0.0019 (10)
C45	0.0157 (11)	0.0193 (12)	0.0226 (12)	0.0001 (9)	0.0074 (9)	0.0015 (10)
C46	0.0400 (15)	0.0125 (12)	0.0517 (17)	0.0016 (11)	0.0212 (13)	0.0049 (11)
C47	0.0215 (12)	0.0272 (14)	0.0497 (17)	0.0056 (11)	0.0158 (12)	−0.0038 (12)
C48	0.0275 (13)	0.0165 (12)	0.0326 (14)	0.0001 (10)	0.0113 (11)	0.0024 (10)

### Geometric parameters (Å, °)

**Table:**

O1—C8	1.361 (3)	C14—H14	0.9500
O1—C9	1.379 (2)	C15—C16	1.380 (3)
O2—C8	1.216 (2)	C16—C17	1.398 (3)
O3—C1	1.353 (3)	C16—H16	0.9500
O3—C10	1.431 (3)	C17—C21	1.382 (3)
O4—C3	1.371 (2)	C17—C18	1.446 (3)
O4—C11	1.418 (3)	C18—C19	1.340 (3)
O5—C12	1.407 (3)	C18—H18	0.9500
O5—H5	0.8400	C19—C20	1.463 (3)
O6—C20	1.363 (3)	C19—C24	1.496 (3)
O6—C21	1.379 (2)	C22—H22A	0.9800
O7—C20	1.216 (2)	C22—H22B	0.9800
O8—C13	1.360 (3)	C22—H22C	0.9800
O8—C22	1.435 (2)	C23—H23A	0.9800
O9—C15	1.368 (2)	C23—H23B	0.9800
O9—C23	1.423 (3)	C23—H23C	0.9800
O10—C24	1.413 (3)	C24—H24A	0.9900
O10—H10	0.8400	C24—H24B	0.9900
O11—C32	1.363 (2)	C25—C26	1.371 (3)
O11—C33	1.375 (2)	C25—C33	1.405 (3)
O12—C32	1.220 (2)	C26—C27	1.397 (3)
O13—C25	1.350 (2)	C26—H26	0.9500
O13—C34	1.438 (3)	C27—C28	1.373 (3)
O14—C27	1.371 (2)	C28—C29	1.405 (3)
O14—C35	1.427 (3)	C28—H28	0.9500
O15—C36	1.414 (3)	C29—C33	1.387 (3)
O15—H15	0.8400	C29—C30	1.442 (3)
O16—C44	1.360 (3)	C30—C31	1.337 (3)
O16—C45	1.375 (2)	C30—H30	0.9500
O17—C44	1.227 (2)	C31—C32	1.448 (3)
O18—C37	1.354 (3)	C31—C36	1.501 (3)
O18—C46	1.434 (3)	C34—H34A	0.9800
O19—C39	1.370 (2)	C34—H34B	0.9800
O19—C47	1.428 (3)	C34—H34C	0.9800
O20—C48	1.412 (3)	C35—H35A	0.9800
O20—H20	0.8400	C35—H35B	0.9800
C1—C2	1.390 (3)	C35—H35C	0.9800
C1—C9	1.395 (3)	C36—H36A	0.9900
C2—C3	1.394 (3)	C36—H36B	0.9900
C2—H2	0.9500	C37—C38	1.374 (3)
C3—C4	1.380 (3)	C37—C45	1.404 (3)
C4—C5	1.396 (3)	C38—C39	1.399 (3)
C4—H4	0.9500	C38—H38	0.9500
C5—C9	1.390 (3)	C39—C40	1.382 (3)
C5—C6	1.440 (3)	C40—C41	1.407 (3)
C6—C7	1.342 (3)	C40—H40	0.9500
C6—H6	0.9500	C41—C45	1.381 (3)
C7—C8	1.461 (3)	C41—C42	1.445 (3)
C7—C12	1.501 (3)	C42—C43	1.346 (3)
C10—H10A	0.9800	C42—H42	0.9500
C10—H10B	0.9800	C43—C44	1.447 (3)
C10—H10C	0.9800	C43—C48	1.501 (3)
C11—H11A	0.9800	C46—H46A	0.9800
C11—H11B	0.9800	C46—H46B	0.9800
C11—H11C	0.9800	C46—H46C	0.9800
C12—H12A	0.9900	C47—H47A	0.9800
C12—H12B	0.9900	C47—H47B	0.9800
C13—C14	1.377 (3)	C47—H47C	0.9800
C13—C21	1.396 (3)	C48—H48A	0.9900
C14—C15	1.398 (3)	C48—H48B	0.9900
			
C8—O1—C9	121.02 (17)	H23A—C23—H23C	109.5
C1—O3—C10	116.84 (16)	H23B—C23—H23C	109.5
C3—O4—C11	118.93 (18)	O10—C24—C19	109.10 (17)
C12—O5—H5	109.5	O10—C24—H24A	109.9
C20—O6—C21	121.18 (17)	C19—C24—H24A	109.9
C13—O8—C22	116.95 (17)	O10—C24—H24B	109.9
C15—O9—C23	118.58 (18)	C19—C24—H24B	109.9
C24—O10—H10	109.5	H24A—C24—H24B	108.3
C32—O11—C33	121.44 (17)	O13—C25—C26	126.4 (2)
C25—O13—C34	115.63 (16)	O13—C25—C33	115.32 (18)
C27—O14—C35	116.87 (17)	C26—C25—C33	118.3 (2)
C36—O15—H15	109.5	C25—C26—C27	120.6 (2)
C44—O16—C45	121.41 (17)	C25—C26—H26	119.7
C37—O18—C46	115.62 (16)	C27—C26—H26	119.7
C39—O19—C47	117.11 (17)	O14—C27—C28	125.0 (2)
C48—O20—H20	109.5	O14—C27—C26	113.80 (19)
O3—C1—C2	125.7 (2)	C28—C27—C26	121.25 (19)
O3—C1—C9	115.42 (18)	C27—C28—C29	119.1 (2)
C2—C1—C9	118.9 (2)	C27—C28—H28	120.5
C1—C2—C3	119.7 (2)	C29—C28—H28	120.5
C1—C2—H2	120.2	C33—C29—C28	119.2 (2)
C3—C2—H2	120.2	C33—C29—C30	116.64 (19)
O4—C3—C4	116.0 (2)	C28—C29—C30	124.2 (2)
O4—C3—C2	122.7 (2)	C31—C30—C29	121.9 (2)
C4—C3—C2	121.3 (2)	C31—C30—H30	119.1
C3—C4—C5	119.5 (2)	C29—C30—H30	119.1
C3—C4—H4	120.3	C30—C31—C32	119.8 (2)
C5—C4—H4	120.3	C30—C31—C36	124.6 (2)
C9—C5—C4	119.2 (2)	C32—C31—C36	115.61 (18)
C9—C5—C6	117.25 (19)	O12—C32—O11	116.3 (2)
C4—C5—C6	123.5 (2)	O12—C32—C31	125.2 (2)
C7—C6—C5	121.3 (2)	O11—C32—C31	118.46 (18)
C7—C6—H6	119.4	O11—C33—C29	121.81 (19)
C5—C6—H6	119.4	O11—C33—C25	116.60 (19)
C6—C7—C8	119.8 (2)	C29—C33—C25	121.59 (19)
C6—C7—C12	125.1 (2)	O13—C34—H34A	109.5
C8—C7—C12	115.05 (18)	O13—C34—H34B	109.5
O2—C8—O1	116.6 (2)	H34A—C34—H34B	109.5
O2—C8—C7	124.7 (2)	O13—C34—H34C	109.5
O1—C8—C7	118.68 (18)	H34A—C34—H34C	109.5
O1—C9—C5	121.96 (19)	H34B—C34—H34C	109.5
O1—C9—C1	116.60 (19)	O14—C35—H35A	109.5
C5—C9—C1	121.44 (19)	O14—C35—H35B	109.5
O3—C10—H10A	109.5	H35A—C35—H35B	109.5
O3—C10—H10B	109.5	O14—C35—H35C	109.5
H10A—C10—H10B	109.5	H35A—C35—H35C	109.5
O3—C10—H10C	109.5	H35B—C35—H35C	109.5
H10A—C10—H10C	109.5	O15—C36—C31	109.18 (17)
H10B—C10—H10C	109.5	O15—C36—H36A	109.8
O4—C11—H11A	109.5	C31—C36—H36A	109.8
O4—C11—H11B	109.5	O15—C36—H36B	109.8
H11A—C11—H11B	109.5	C31—C36—H36B	109.8
O4—C11—H11C	109.5	H36A—C36—H36B	108.3
H11A—C11—H11C	109.5	O18—C37—C38	126.0 (2)
H11B—C11—H11C	109.5	O18—C37—C45	115.65 (18)
O5—C12—C7	109.23 (17)	C38—C37—C45	118.3 (2)
O5—C12—H12A	109.8	C37—C38—C39	120.7 (2)
C7—C12—H12A	109.8	C37—C38—H38	119.7
O5—C12—H12B	109.8	C39—C38—H38	119.7
C7—C12—H12B	109.8	O19—C39—C40	125.1 (2)
H12A—C12—H12B	108.3	O19—C39—C38	113.91 (19)
O8—C13—C14	125.7 (2)	C40—C39—C38	121.0 (2)
O8—C13—C21	115.18 (18)	C39—C40—C41	118.8 (2)
C14—C13—C21	119.1 (2)	C39—C40—H40	120.6
C13—C14—C15	120.1 (2)	C41—C40—H40	120.6
C13—C14—H14	119.9	C45—C41—C40	119.5 (2)
C15—C14—H14	119.9	C45—C41—C42	117.00 (19)
O9—C15—C16	116.2 (2)	C40—C41—C42	123.5 (2)
O9—C15—C14	123.4 (2)	C43—C42—C41	121.5 (2)
C16—C15—C14	120.4 (2)	C43—C42—H42	119.3
C15—C16—C17	119.9 (2)	C41—C42—H42	119.3
C15—C16—H16	120.0	C42—C43—C44	119.4 (2)
C17—C16—H16	120.0	C42—C43—C48	124.3 (2)
C21—C17—C16	119.1 (2)	C44—C43—C48	116.22 (18)
C21—C17—C18	117.25 (19)	O17—C44—O16	116.2 (2)
C16—C17—C18	123.7 (2)	O17—C44—C43	124.9 (2)
C19—C18—C17	121.5 (2)	O16—C44—C43	118.83 (18)
C19—C18—H18	119.3	O16—C45—C41	121.82 (19)
C17—C18—H18	119.3	O16—C45—C37	116.53 (19)
C18—C19—C20	119.5 (2)	C41—C45—C37	121.6 (2)
C18—C19—C24	125.2 (2)	O18—C46—H46A	109.5
C20—C19—C24	115.25 (18)	O18—C46—H46B	109.5
O7—C20—O6	116.7 (2)	H46A—C46—H46B	109.5
O7—C20—C19	124.6 (2)	O18—C46—H46C	109.5
O6—C20—C19	118.63 (19)	H46A—C46—H46C	109.5
O6—C21—C17	121.93 (19)	H46B—C46—H46C	109.5
O6—C21—C13	116.71 (19)	O19—C47—H47A	109.5
C17—C21—C13	121.4 (2)	O19—C47—H47B	109.5
O8—C22—H22A	109.5	H47A—C47—H47B	109.5
O8—C22—H22B	109.5	O19—C47—H47C	109.5
H22A—C22—H22B	109.5	H47A—C47—H47C	109.5
O8—C22—H22C	109.5	H47B—C47—H47C	109.5
H22A—C22—H22C	109.5	O20—C48—C43	109.20 (16)
H22B—C22—H22C	109.5	O20—C48—H48A	109.8
O9—C23—H23A	109.5	C43—C48—H48A	109.8
O9—C23—H23B	109.5	O20—C48—H48B	109.8
H23A—C23—H23B	109.5	C43—C48—H48B	109.8
O9—C23—H23C	109.5	H48A—C48—H48B	108.3
			
C10—O3—C1—C2	−6.8 (3)	C34—O13—C25—C26	−3.1 (3)
C10—O3—C1—C9	173.8 (2)	C34—O13—C25—C33	176.2 (2)
O3—C1—C2—C3	179.7 (2)	O13—C25—C26—C27	179.2 (2)
C9—C1—C2—C3	−0.9 (3)	C33—C25—C26—C27	0.0 (3)
C11—O4—C3—C4	174.6 (2)	C35—O14—C27—C28	1.5 (3)
C11—O4—C3—C2	−6.6 (3)	C35—O14—C27—C26	−178.6 (2)
C1—C2—C3—O4	−179.2 (2)	C25—C26—C27—O14	179.78 (19)
C1—C2—C3—C4	−0.4 (4)	C25—C26—C27—C28	−0.3 (3)
O4—C3—C4—C5	−179.9 (2)	O14—C27—C28—C29	−179.8 (2)
C2—C3—C4—C5	1.2 (4)	C26—C27—C28—C29	0.2 (3)
C3—C4—C5—C9	−0.7 (3)	C27—C28—C29—C33	0.1 (3)
C3—C4—C5—C6	178.3 (2)	C27—C28—C29—C30	−179.5 (2)
C9—C5—C6—C7	0.4 (3)	C33—C29—C30—C31	0.6 (3)
C4—C5—C6—C7	−178.6 (2)	C28—C29—C30—C31	−179.8 (2)
C5—C6—C7—C8	−1.3 (3)	C29—C30—C31—C32	−0.6 (3)
C5—C6—C7—C12	177.8 (2)	C29—C30—C31—C36	−180.0 (2)
C9—O1—C8—O2	−179.01 (19)	C33—O11—C32—O12	179.87 (19)
C9—O1—C8—C7	0.9 (3)	C33—O11—C32—C31	−0.9 (3)
C6—C7—C8—O2	−179.4 (2)	C30—C31—C32—O12	179.9 (2)
C12—C7—C8—O2	1.4 (3)	C36—C31—C32—O12	−0.7 (3)
C6—C7—C8—O1	0.7 (3)	C30—C31—C32—O11	0.7 (3)
C12—C7—C8—O1	−178.54 (19)	C36—C31—C32—O11	−179.87 (19)
C8—O1—C9—C5	−1.8 (3)	C32—O11—C33—C29	1.0 (3)
C8—O1—C9—C1	178.91 (19)	C32—O11—C33—C25	−179.04 (19)
C4—C5—C9—O1	−179.8 (2)	C28—C29—C33—O11	179.65 (19)
C6—C5—C9—O1	1.1 (3)	C30—C29—C33—O11	−0.8 (3)
C4—C5—C9—C1	−0.6 (3)	C28—C29—C33—C25	−0.3 (3)
C6—C5—C9—C1	−179.6 (2)	C30—C29—C33—C25	179.2 (2)
O3—C1—C9—O1	0.1 (3)	O13—C25—C33—O11	1.0 (3)
C2—C1—C9—O1	−179.35 (19)	C26—C25—C33—O11	−179.67 (19)
O3—C1—C9—C5	−179.2 (2)	O13—C25—C33—C29	−179.0 (2)
C2—C1—C9—C5	1.4 (3)	C26—C25—C33—C29	0.3 (3)
C6—C7—C12—O5	1.1 (3)	C30—C31—C36—O15	1.5 (3)
C8—C7—C12—O5	−179.77 (19)	C32—C31—C36—O15	−177.92 (19)
C22—O8—C13—C14	3.7 (3)	C46—O18—C37—C38	1.2 (3)
C22—O8—C13—C21	−176.6 (2)	C46—O18—C37—C45	−178.6 (2)
O8—C13—C14—C15	−179.2 (2)	O18—C37—C38—C39	−179.2 (2)
C21—C13—C14—C15	1.1 (3)	C45—C37—C38—C39	0.5 (3)
C23—O9—C15—C16	−172.5 (2)	C47—O19—C39—C40	−1.8 (3)
C23—O9—C15—C14	8.5 (3)	C47—O19—C39—C38	178.3 (2)
C13—C14—C15—O9	179.2 (2)	C37—C38—C39—O19	−179.7 (2)
C13—C14—C15—C16	0.3 (4)	C37—C38—C39—C40	0.4 (4)
O9—C15—C16—C17	179.6 (2)	O19—C39—C40—C41	179.6 (2)
C14—C15—C16—C17	−1.4 (4)	C38—C39—C40—C41	−0.5 (3)
C15—C16—C17—C21	1.1 (3)	C39—C40—C41—C45	−0.2 (3)
C15—C16—C17—C18	−178.9 (2)	C39—C40—C41—C42	179.5 (2)
C21—C17—C18—C19	−1.0 (3)	C45—C41—C42—C43	0.1 (3)
C16—C17—C18—C19	179.1 (2)	C40—C41—C42—C43	−179.6 (2)
C17—C18—C19—C20	0.6 (3)	C41—C42—C43—C44	−1.1 (3)
C17—C18—C19—C24	−179.4 (2)	C41—C42—C43—C48	178.2 (2)
C21—O6—C20—O7	178.95 (19)	C45—O16—C44—O17	−179.98 (19)
C21—O6—C20—C19	−1.2 (3)	C45—O16—C44—C43	0.0 (3)
C18—C19—C20—O7	−179.7 (2)	C42—C43—C44—O17	−179.0 (2)
C24—C19—C20—O7	0.3 (3)	C48—C43—C44—O17	1.6 (3)
C18—C19—C20—O6	0.4 (3)	C42—C43—C44—O16	1.1 (3)
C24—C19—C20—O6	−179.6 (2)	C48—C43—C44—O16	−178.3 (2)
C20—O6—C21—C17	0.8 (3)	C44—O16—C45—C41	−1.0 (3)
C20—O6—C21—C13	−179.22 (19)	C44—O16—C45—C37	178.65 (19)
C16—C17—C21—O6	−179.81 (19)	C40—C41—C45—O16	−179.30 (19)
C18—C17—C21—O6	0.2 (3)	C42—C41—C45—O16	1.0 (3)
C16—C17—C21—C13	0.2 (3)	C40—C41—C45—C37	1.1 (3)
C18—C17—C21—C13	−179.7 (2)	C42—C41—C45—C37	−178.7 (2)
O8—C13—C21—O6	−1.0 (3)	O18—C37—C45—O16	−1.1 (3)
C14—C13—C21—O6	178.70 (19)	C38—C37—C45—O16	179.12 (19)
O8—C13—C21—C17	178.9 (2)	O18—C37—C45—C41	178.5 (2)
C14—C13—C21—C17	−1.3 (3)	C38—C37—C45—C41	−1.2 (3)
C18—C19—C24—O10	3.5 (3)	C42—C43—C48—O20	1.2 (3)
C20—C19—C24—O10	−176.47 (19)	C44—C43—C48—O20	−179.46 (19)

### Hydrogen-bond geometry (Å, °)

**Table:**

*D*—H···*A*	*D*—H	H···*A*	*D*···*A*	*D*—H···*A*
O20—H20···O2^i^	0.84	2.00	2.829 (2)	171
O15—H15···O12^ii^	0.84	1.96	2.800 (2)	175
O10—H10···O7^iii^	0.84	2.00	2.838 (2)	174
O5—H5···O17^iv^	0.84	1.96	2.790 (2)	173

Symmetry codes: (i) *x*, −*y*+1/2, *z*−1/2; (ii) −*x*, *y*−1/2, −*z*+1/2; (iii) −*x*+1, *y*+1/2, −*z*+1/2; (iv) *x*, −*y*−1/2, *z*+1/2.
